# Exogenously applied growth regulators protect the cotton crop from heat-induced injury by modulating plant defense mechanism

**DOI:** 10.1038/s41598-018-35420-5

**Published:** 2018-11-20

**Authors:** Muhammad Sarwar, Muhammad Farrukh Saleem, Najeeb Ullah, Muhammad Rizwan, Shafaqat Ali, Muhammad Rizwan Shahid, Saud A. Alamri, Mohammed Nasser Alyemeni, Parvaiz Ahmad

**Affiliations:** 1 Agronomic Research Institute, Ayub Agricultural Research Institute, Faisalabad, Pakistan; 2 Department of Agronomy, University of Agriculture Faisalabad, Faisalabad, Pakistan; 3 Queensland Alliance for Agriculture and Food Innovation, Centre for Plant Science, The University of Queensland Wilsonton Heights, Toowoomba, QLD 4350 Australia; 4 Department of Environmental Sciences and Engineering, Government College University Allama Iqbal Road, 38000 Faisalabad, Pakistan; 5 Institute of Soil and Environmental Sciences, University of Agriculture, Faisalabad, 38000 Pakistan; 6 Department of Botany and Microbiology, College of Science, King Saud University, Riyadh, Saudi Arabia; 7 Department of Botany, S.P. College, Maulana Azad Road, Srinagar, Jammu and Kashmir 190001 India

## Abstract

Episodes of extremely high temperature during reproductive stages of cotton crops are common in many parts of the world. Heat stress negatively influences plant growth, physiology and ultimately lint yield. This study attempts to modulate heat-induced damage to cotton crops via application of growth regulators e.g. hydrogen peroxide (H_2_O_2_ 30ppm), salicylic acid (SA 50ppm), *moringa* leaf extract (MLE 30 times diluted) and ascorbic acid (ASA 70ppm). Cotton plants were exposed to different thermal regimes by staggering sowing time (field) or exposing to elevated temperatures (38/24 °C and 45/30 °C) for one week during reproductive growth stages (glasshouse). Elevated temperatures significantly induced lipid membrane damage, which was evident from an increased malondialdehyde (MDA) level in cotton leaves. Heat-stressed plants also experienced a significant reduction in leaf chlorophyll contents, net photosynthetic rate and lint yield. Hydrogen peroxide outclassed all the other regulators in increasing leaf SOD, CAT activity, chlorophyll contents, net photosynthetic rate, number of sympodial branches, boll weight and fiber quality components. For example, hydrogen peroxide improved boll weight of heat stressed plants by 32% (supra), 12% (sub) under glasshouse and 18% (supra) under field conditions compared with water treated plants under the same temperatures. Growth regulators, specifically, H_2_O_2_ protected physiological processes of cotton from heat-induced injury by capturing reactive oxygen species and modulating antioxidant enzymes. Thus, cotton performance in the future warmer climates may be improved through regulation (endogenous) or application (exogenous) hormones during reproductive phases.

## Introduction

Abiotic stresses such as salinity, drought and extreme temperatures strongly influence crop production throughout the world. Among these, high temperature is one of the most common stresses, which impacts defensive system, chlorophyll contents, photosynthetic apparatus, antioxidant enzymes and productivity of field crops^1^. For example, daily average temperature for optimum cotton growth ranges from 27–29 °C and 25.5 °C^2,3^ and cotton crop is predominantly cultivated under semiarid conditions where maximum day temperature reaches to 48–50 °C^4^. Important physiological process e.g. leaf chlorophyll and carbon assimilation process of cotton has been found very sensitive to high temperature^5,6^. For example, a small increase in air temperature above optimum (30 °C) can significantly reduce leaf photosynthesis^7^. Further, a temperature above 35–40 °C significantly restrict elongation of fruiting branch (sympodial branches) in cotton^2^. Heat stress influences cellular biochemistry by accelerating reactive oxygen species (ROS) production^8^. While, plants may metabolize these ROS through up-regulating antioxidants defense enzymes e.g. superoxide dismutase (SOD) and catalase (CAT)^9,10^.

Plant growth regulators have been found effective in improving crop performance against abiotic stresses by activating defensive system^11^. For example, hydrogen peroxide, a signaling molecule has been found effective to activate plant defensive system in plants under high temperature stress^12^. Similarly, ascorbic acid can reduce oxidative cellular injury^13^, improve chlorophyll contents and maintain redox state of photosynthetic process^14,15^. An organic extract from *Moringa* leaves containing zeatine-a group of cytokinin^16^ has also been found effective in increasing chlorophyll of drought-stressed maize crop^17^.

As the physiological process of plants including carbon assimilation are very sensitive to heat stress in cotton, we proposed that negative effects of heat could be meliorated by modulating plant defense mechanism through plant growth regulators. For this, we conducted experiments under glasshouse and field conditions to (1) Compare the leaf bio-chemistry, physiology, yield components and fiber quality of cotton under different temperature regimes and (2) To assess the effect of various growth substances for mitigating the adverse effects of high temperature.

## Materials and Methods

### Glasshouse experiment

Glasshouse of Faculty of Agriculture, University of Agriculture Faisalabad (UAF), Pakistan was used to conduct the pot trials. Cotton plants were grown under 14/10 h day/night light. To get a clear response to growth regulators, a medium heat tolerant variety (cv. AA-802), was used in these experiments. Soil properties and growth condition were same as have been reported in the previous study^18^. Three temperature regimes viz. 45/30 °C (prevails in Pakistan during cotton growing season), 38/24 °C (considered as critical range for cotton) and 32/20 °C (optimum) were maintained under three different chambers of glass house. Florescent bulbs were lighted in these chambers as supplement light as the main part of photosynthetically active radiations (1400–1600 mmol m^−2^ sec^−1^) were available from sun. Different foliar sprays (distilled water, 30 ppm hydrogen peroxide, 50 ppm salicylic acid, 30 times diluted *moringa* leaf extract and 70 ppm solution of ascorbic acid) were applied on cotton plants and pots were shifted after 24 hours of foliar spray to medium and high temperature chambers. Seven days after this foliar spray, leaves samples were taken to measure chlorophyll contents and the activity of superoxide dismutase (SOD) and catalase (CAT) enzymes and pots were shifted back to optimum temperature range chamber. It took 120 days to complete the experiment.

### Conditions for field experiment

#### Location

The field experiment was conducted at Agronomy Farm in 2012 and repeated in 2013. During first year, crop was sown on 2^nd^ April, 3^rd^ May and 17^th^ June while during second year planting was done on 4^th^ April, 2^nd^ May and 19^th^ June. Weather data were obtained from the staff engaged at the metrological observatory of Agronomy Department, University of Agriculture Faisalabad, Pakistan and has been shown in previous study^18^. Different sowing times referred as thermal regimes provided different temperatures to crop^19^. Randomized complete block design with split plot treatment structure was employed keeping sowing dates in main plot and growth substances in subplots. Six lines were sown in each experimental unit measuring 6.0 m × 4.5 m. Foliar spray of 30 ppm hydrogen peroxide, 50 ppm salicylic acid, 30 times diluted *moringa* leaf extract and 70 ppm of ascorbic acid were applied thrice at square initiation, flower appearance and at boll initiation. Seven days after this foliar spray, leaves samples were taken to measure chlorophyll contents and the activity of superoxide dismutase (SOD) and catalase (CAT) enzymes.

#### Leaf physiology

In order to measure leaf SOD activity, photoreduction of nitroblue tetrazolium was inhibited and the absorbance was recorded at 470 nm^20^. A total volume of 3 mL of reaction mixture (50 mM phosphate buffer (pH 7) + 5.9 mMH_2_O_2_) was used to extract CAT activity from 0.1 mL enzyme extract^21^. Procedure of^22^ was used to measure leaf MDA contents.

Infrared gas analyzer (LCi Analyser having Broad Head, Part Number LCi-002/B with Serial Number 32455) was used to determine the net photosynthetic rate (Pn, µ mol m^−2^sec^−1^) at three different growth stages of the cotton crop.

#### Fiber quality

Seed cotton from ten randomized selected plants were picked. After picking, 10 g of lint sample was kept at 20 °C with 65–68% relative humidity for 6–8 hours for conditioning purpose. High volume instrument (HVI) was used to measure fiber length (mm), fineness/micronaire and strength (g/tex).

#### Moringa leaf extract (MLE), boll weight (g) and number of sympodial branches/plants

Fully grown *moringa* leaves (*Moringa oleifera*) were collected to obtain their extract.

Cotton bolls were collected from the 10 mature plants of each experimental unit, and average boll weight was determined by dividing total seed cotton yield per plant to the total number of bolls. Number of sympodial branches per plant were calculated from 10 randomized selected plants of each experimental unit.

#### Statistical procedures

Data were analyzed through Statistix 10.1 software. Individual and interactive effects of high temperature and growth regulators on leaf SOD, CAT, chlorophyll contents, number of sympodial branches per plant, averaged boll weight (g) and fiber quality components were analyzed through two-way analysis of variance (ANOVA). Regression analysis (R^2^) was run separately for all studied parameters under field and glass house conditions. Level of significance was reported at 5 and 1% of probability.

## Results

### Green house experiment

**Figure 1:**
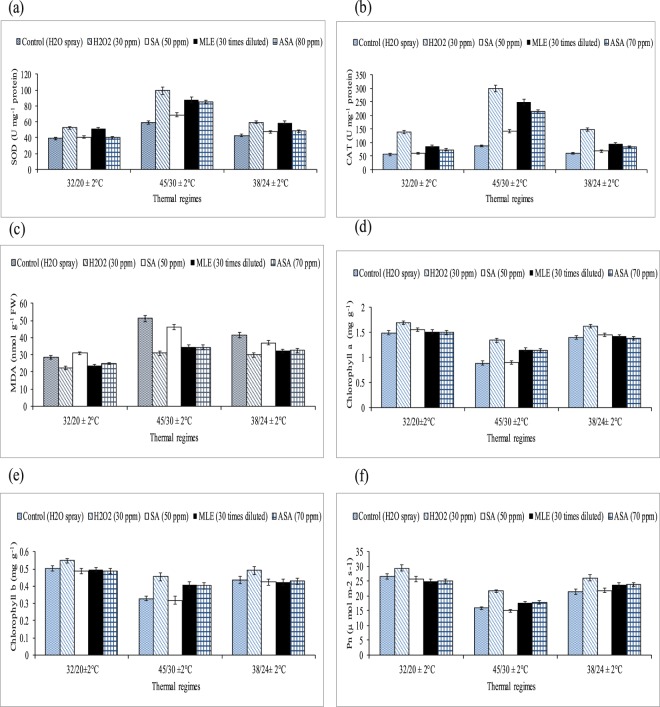
Effect of growth regulators and thermal regimes on (**a**) superoxide dismutase (SOD), (**b**) catalase (CAT), (**c**) malondialdehyde contents (nmol g^−1^ FW), (**d**,**e**) chlorophyll contents and (**f**) net photosynthetic rate-Pn (µ mol m^−2^ sec^−1^) in glasshouse conditions. Bars indicate SD and mean values with ± SD (n = 3) are given.

**Figure 2:**
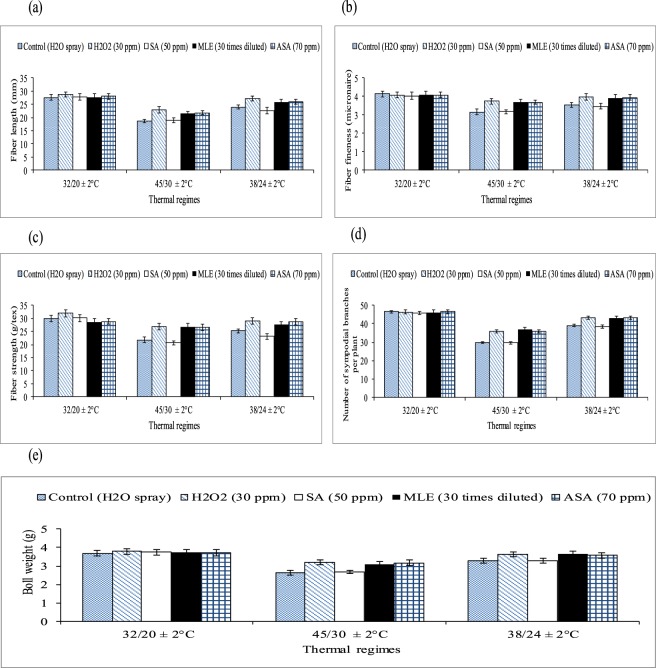
Effect of growth regulators and thermal regimes on (**a**–**c**) fiber quality components, (**d**) number of sympodial branches per plant and (**e**) boll weight under glass house condition. Bars indicate SD and mean values with ± SD (n = 3) are given.

Medium (38/24 °C ± 2) and high temperature (45/30 °C ± 2) stress at three reproductive stages significantly enhanced MDA contents and antioxidant enzyme levels (SOD and CAT) of cotton leaves. Heat-stressed cotton plants had significantly lower leaf chlorophyll contents, net photosynthetic rate, fiber quality (fiber length, fineness and strength), number of sympodial branches per plant and averaged boll weight (g) (Tables 1–2, Figs 1 and 2). In the absence of growth regulator application, heat-stressed plants (exposed to 45/30 °C) had 0.49, 1.40 and 0.78 times greater (averaged across three growth/reproductive phases) SOD, CAT and MDA contents over the optimal temperature plants. Application of growth substances e.g. H_2_O_2_ followed by ASA and MLE further increased antioxidant enzymes but reduced MDA contents in cotton leaves at three reproductive stages than SA and water treated plants. For example, H_2_O_2_ treated leaves had 0.69, 0.38 and 0.34 folds higher SOD contents than leaves treated with water under supra, sub and optimal temperature regimes, respectively (averaged across of three growth/reproductive phases). Similarly, H_2_O_2_ enhanced chlorophyll a contents in cotton leaves by 50%, 15% and 13% under supra (high), sub (medium) and optimal temperature regimes, respectively than the leaves treated with water.

**Table 1:** Effect of growth regulators and thermal regimes on superoxide dismutase (SOD), catalase (CAT), chlorophyll contents and malondialdehyde contents (nmol g^−1^ FW) in glass house conditions.

Thermal regimes	Regulators	SOD	CAT	MDA (nmol g^−1^ FW)	Chlorophyll a	Chlorophyll b
32/20 °C	Control	39.21 c ± 0.88	56.37 d ± 2.65	28.69 a ± 1.6	1.49 b ± 0.80	0.50 b ± 0.30
	H_2_O_2_ (30 ppm)	52.59 a ± 0.50	139.13 a ± 7.60	22.20 b ± 1.1	1.70 a ± 1.20	0.55 a ± 0.32
	SA (70 ppm)	40.46 c ± 0.90	59.50 d ± 3.35	29.97 a ± 1.8	1.53 b ± 0.93	0.49 b ± 0.28
	MLE (30 times diluted)	51.57 a ± 0.79	84.90 b ± 5.91	23.50 b ± 1.2	1.51 b ± 1.26	0.50 b ± 0.26
	ASA (70 ppm)	45.94 b ± 0.79	72.84 c ± 3.98	24.85 bc ± 1.3	1.50 b ± 1.16	0.49 b ± 0.24
45/30 °C	Control	58.82 d ± 1.20	87.33 e ± 4.57	51.16 a ± 2.6	0.89 c ± 0.56	0.33 c ± 0.19
	H_2_O_2_ (30 ppm)	99.24 a ± 2.55	300.31 a ± 16.44	30.88 d ± 1.7	1.34 a ± 0.83	0.45 a ± 0.21
	SA (70 ppm)	68.93 c ± 0.55	142.17 d ± 7.53	48.06 b ± 2.5	0.90 c ± 0.60	0.32 c ± 0.22
	MLE (30 times diluted)	88.09 b ± 2.08	249.97 b ± 13.18	34.58 c ± 1.6	1.15 b ± 0.70	0.41 b ± 0.25
	ASA (70 ppm)	86.38 b ± 1.20	214.03 c ± 11.80	34.45 c ± 1.7	1.14 b ± 0.66	0.40 b ± 0.22
38/24 °C	Control	42.90 d ± 0.87	60.83 e ± 3.89	41.51 a ± 2.2	1.40 b ± 0.78	0.44 b ± 0.23
	H_2_O_2_ (30 ppm)	59.34 a ± 1.04	147.74 a ± 8.18	30.01 d ± 1.6	1.62 a ± 0.91	0.49 a ± 0.27
	SA (70 ppm)	47.14 c ± 1.05	67.91 d ± 4.10	38.01 b ± 1.9	1.44 b ± 0.82	0.42 b ± 0.18
	MLE (30 times diluted)	59.00 a ± 0.97	94.55 b ± 5.85	32.19 c ± 1.6	1.43 b ± 0.76	0.43 b ± 0.21
	ASA (70 ppm)	53.16 b ± 0.94	84.41 c ± 5.36	32.46 c ± 1.7	1.39 b ± 0.85	0.43 b ± 0.24
	LSD	2.83	5.31	1.57	0.051	0.023

Mean of three replications (n = 3) ± SE and variants having same alphabets are not statistically significant at *P˂ 0.05*. Main factors and interaction are significant at *P˂ 0.01*. By using the LSD of the interaction between growth regulators and thermal regimes, lettering is done separately for each thermal regime.

**Table 2:** Effect of growth regulators and thermal regimes on net photosynthetic rate-Pn (µ mol m^−2^ sec^−1^), fiber quality components, number of sympodial branches per plant and boll weight (g) under glass house conditions.

Thermal regimes	Regulators	Pn	Fiber fineness (micronaire)	Fiber length (mm)	Fiber strength (g/tex)	No. of sympodial branches per plant	Boll weight (g)
32/20 °C	H_2_O_2_ (30 ppm)	28.4 ± 0.53 a	4.0 a ± 0.20	28.7 a ± 1.5	30.9 a ± 1.7	23.3 a ± 0.56	3.7 a ± 0.19
	SA (70 ppm)	25.6 ± 0.50 b	3.9 a ± 0.19	27.8 a ± 1.3	30.1 a ± 1.6	22.8 a ± 0.44	3.8 a ± 0.20
	MLE (30 times diluted)	24.9 ± 0.50 b	4.2 a ± 0.23	27.8 a ± 1.6	29.7 a ± 1.4	23.2 a ± 0.41	3.8 a ± 0.21
	ASA (70 ppm)	25.1 ± 0.41 b	4.1 a ± 0.21	28.1 a ± 1.4	29.8 a ± 1.5	22.9 a ± 0.40	3.7 a ± 0.18
45/30 °C	Control	21.5 ± 0.52 c	3.2 b ± 0.16	18.6 b ± 1.0	21.8 b ± 1.1	14.8 b ± 0.31	2.6 b ± 0.17
	H_2_O_2_ (30 ppm)	26.1 ± 0.49 a	3.8 a ± 0.19	23.0 a ± 1.1	26.9 a ± 1.3	17.6 a ± 0.37	3.4 a ± 0.14
	SA (70 ppm)	21.9 ± 0.46 c	3.2 b ± 0.16	18.8 b ± 0.9	20.7 b ± 1.0	14.5 b ± 0.33	2.7 b ± 0.16
	MLE (30 times diluted)	23.7 ± 0.49 b	3.7 a ± 0.18	21.6 a ± 1.2	26.8 a ± 1.2	17.4 a ± 0.43	3.2 a ± 0.13
	ASA (70 ppm)	24.0 ± 0.39 b	3.7 a ± 0.17	21.8 a ± 1.3	25.7 a ± 1.3	17.3 a ± 0.31	3.1 a ± 0.15
38/24 °C	Control	15.9 ± 0.31 c	3.5 b ± 0.18	23.9 c ± 1.4	25.2 b ± 1.1	19.7 b ± 0.50	3.2 a ± 0.17
	H_2_O_2_ (30 ppm)	21.6 ± 0.36 a	4.0 a ± 0.21	27.1 a ± 1.5	29.0 a ± 1.5	22.1 a ± 0.45	3.6 b ± 0.18
	SA (70 ppm)	15.0 ± 0.29 c	3.4 b ± 0.17	22.6 c ± 1.1	24.1 b ± 1.2	19.7 b ± 0.39	3.3 a ± 0.19
	MLE (30 times diluted)	17.8 ± 0.32 b	3.9 a ± 0.23	25.8 b ± 1.2	27.7 a ± 1.4	22.9 a ± 0.53	3.3 b ± 0.16
	ASA (70 ppm)	17.9 ± 0.37 b	3.9 a ± 0.20	26.0 a ± 1.4	28.6 a ± 1.6	22.8 a ± 0.52	3.7 a ± 0.19
	LSD	1.42	0.21	1.43	1.51	1.02	0.20

Mean of three replications (n = 3) ± SE and variants having same alphabets are not statistically significant at *P˂ 0.05*. Main factors and interaction are significant at *P˂ 0.01*. By using the LSD of the interaction between growth regulators and thermal regimes, lettering is done separately for each thermal regime.

Thermal regimes and growth regulators showed significant effect (*P* < 0.01) on Pn, number of sympodial branches per plant and averaged boll weight (Table 2, Figs 1f and 2d–e). Averaged across three reproductive stages, Pn was reduced by 59% (supra-optimal) and 34% (sub-optimal) compared the water treated plants of these thermal regimes with water treated plants of optimal thermal regime. Among the regulators, H_2_O_2_ (followed by ASA and MLE) improved Pn by 32% and 21% (averaged across three growth/reproductive phases) in supra and sub-optimal thermal regime than water spray. Number of sympodial branches per plant were decreased by 40% and 8% (averaged across of under various treatments) in the plants exposed to supra (high) and sub optimal temperature regimes. Various growth substances (H_2_O_2_, MLE and ASA) improved number of sympodial branches per plant significantly under sub and supra-optimal thermal regimes. Among the growth regulators, H_2_O_2_ increased number of sympodial branches by 18.5% and 12% under supra and sub-optimal thermal regimes than the optimal thermal regime. Similarly, boll weight was reduced in medium and high temperature regimes and the foliar spray of hydrogen peroxide improved boll weight. High temperature (sub and supra-optimal thermal regimes) significantly reduced fiber quality components e.g. fiber fineness, fiber length and fiber strength. Different growth substances significantly improved fiber quality components.

### Relationship between leaf MDA, fiber quality and boll weight

The association between MDA with quality parameters (fiber length, fineness and fiber strength) (Fig. 5a–c) and MDA with boll weight was studied through regression analysis (Fig. 5d). The degree of relation differed significantly for optimal (32/20 °C ± 2), sub-optimal (38/24 °C ± 2) and supra-optimal temperature regimes. Irrespective of degree of association, leaf MDA contents were positively and non-significantly correlated with fiber quality parameters (fiber fineness, fiber length and with fiber strength under optimal thermal regime (Figs 5 and 6). The association between MDA contents and fiber quality parameters was significant and negative under sub and supra-optimal thermal regimes, irrespective of magnitude. Mean squares of regression were significant at 1% in supra-optimal and at 5% in sub-optimal thermal regime.

### Field experiment

**Figure 3:**
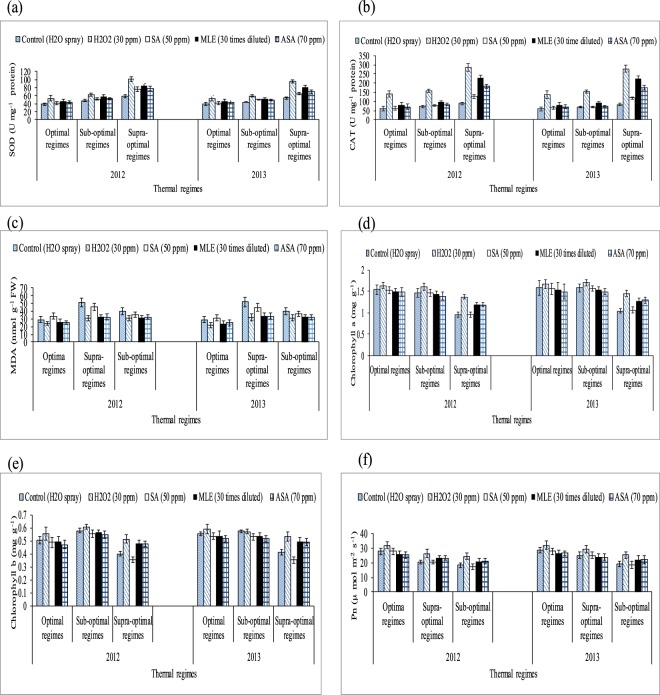
Effect of growth regulators and thermal regimes on (**a**) superoxide dismutase (SOD), (**b**) catalase (CAT), (**c**) malondialdehyde contents (nmol g^−1^ FW), (**d**,**e**) chlorophyll contents and (**f**) net photosynthetic rate-Pn (µ mol m^−2^ sec^−1^) under field conditions during both years of study. Bars indicate SD and mean values with ± SD (n = 3) are given.

Exposure to increasing temperature significantly increased antioxidant enzymes (SOD and CAT) and MDA in cotton leaves (thermal regimes × regulators *P* < 0.05) (Table 3 and Fig. 3a–c). In the water-sprayed plants, leaf SOD activity was increased by 0.44 and 0.17 folds under high temperature-supra and medium temperature-sub-optimal regimes over optimal thermal regime. Similarly, leaf CAT contents were increased by 1.16 folds and 0.14 folds under supra and sub-optimal thermal regime (averaged across both years of study at three reproductive stages). Plants under high temperature regime (May sowing during first and April sowing during second year of the study) showed higher leaf MDA contents as compared to plants grown under optimal thermal regime-June sowing. Hydrogen peroxide-H_2_O_2_, ascorbic acid-ASA and *moringa* leaf extract-MLE stimulated plant defensive system significant than salicylic acid and water treated plants. Hydrogen peroxide treated plants had 0.75, 0.33 and 0.36 folds higher leaf SOD activity over the plants treated with water under high (supra), medium (sub) and optimal temperature regimes, respectively. Similarly, H_2_O_2_ increased CAT contents by 2.26, 1.19 and 1.31 folds under supra (high), sub (medium) and optimal thermal regimes, respectively than plants treated with water.

**Table 3:** Effect of growth regulators and thermal regimes on superoxide dismutase (SOD), catalase (CAT), malondialdehyde contents (nmol g^−1^ FW) and chlorophyll a contents in field conditions during 2012 and 2013.

Thermal regimes	Regulators	SOD 2012	SOD 2013	CAT 2012	CAT 2013	MDA (nmol g^−1^ FW) 2012	MDA (nmol g^−1^ FW) 2013	Chlorophyll a 2012	Chlorophyll a 2013
Optimal regime	Control	39.08 ab ± 2.80	39.47 bc ± 2.69	60.69 b ± 4.42	59.37 b ± 4.00	40.25 a ± 3.8	50.03 a ± 4.9	1.54 a ± 0.11	1.58 a ± 0.11
	H_2_O_2_ (30 ppm)	53.69 a ± 3.84	53.70 a ± 4.57	140.89 a ± 10.78	136.55 a ± 12.10	31.00 c ± 2.9	32.04 c ± 3.1	1.64 a ± 0.14	1.67 a ± 0.13
	SA (70 ppm)	41.13 ab ± 2.52	40.84 ab ± 2.7	62.19 b ± 4.47	66.61 b ± 4.15	35.50 b ± 3.4	44.72 b ± 4.0	1.53 a ± 0.09	1.56 a ± 0.10
	MLE (30 times diluted)	47.03 a ± 3.20	47.14 a ± 3.24	81.10 b ± 6.99	79.47 b ± 6.47	31.25 c ± 2.9	33.56 c ± 3.2	1.50 a ± 0.10	1.54 a ± 0.12
	ASA (70 ppm)	43.99 a ± 2.92	44.28 ab ± 3.04	70.72 b ± 5.02	70.32 b ± 5.72	32.33 c ± 3.1	33.72 c ± 3.3	1.48 a ± 0.09	1.49 ab ± 0.10
Supra-optimal	Control	58.54 d ± 4.87	54.40 d ± 4.12	89.96 e ± 6.50	83.65 e ± 6.74	51.96 a ± 4.8	39.49 a ± 3.8	0.96 c ± 0.07	1.04 c ± 0.07
	H_2_O_2_ (30 ppm)	101.26 a ± 7.83	96.23 a ± 6.12	286.76 a ± 20.36	279.01 a ± 20.28	31.15 c ± 3.0	31.13 b ± 3.0	1.37 a ± 0.09	1.45 a ± 0.11
	SA (70 ppm)	74.69 c ± 5.48	65.58 c ± 3.89	127.02 d ± 9.06	118.74 d ± 8.64	45.58 b ± 4.3	36.33 a ± 3.5	0.95 c ± 0.06	1.06 c ± 0.08
	MLE (30 times diluted)	85.37 b ± 6.07	81.49 b ± 7.27	229.24 b ± 17.04	223.28 b ± 16.41	32.56 c ± 3.1	32.96 ab ± 3.1	1.20 b ± 0.10	1.28 b ± 0.07
	ASA (70 ppm)	87.92 b ± 6.58	79.65 b ± 5.39	182.90 c ± 12.85	175.23 c ± 11.63	32.60 c ± 3.0	32.46 ab ± 2.9	1.19 b ± 0.08	1.29 b ± 0.10
Sub-optimal	Control	48.21 ab ± 3.91	43.97 cd ± 2.71	72.39 c ± 5.70	69.56 bc ± 5.62	29.07 a ± 2.8	28.93 a ± 2.7	1.46 a ± 0.1	1.58 a ± 0.09
	H_2_O_2_ (30 ppm)	63.04 a ± 3.87	59.86 a ± 4.27	157.78 a ± 10.20	153.05 a ± 9.16	24.21 b ± 2.1	22.17 b ± 2.1	1.60 a ± 0.13	1.71 a ± 0.14
	SA (70 ppm)	51.21 ab ± 4.92	51.03 b ± 2.99	74.18 c ± 4.27	68.00 bc ± 4.20	31.67 a ± 3.0	31.04 a ± 3.0	1.47 a ± 0.11	1.57 a ± 0.10
	MLE (30 times diluted)	58.08 a ± 4.01	51.51 b ± 3.20	97.20 b ± 6.70	92.02 b ± 6.83	24.30 b ± 2.2	24.19 b ± 2.3	1.43 ab ± 0.10	1.53 ab ± 0.11
	ASA (70 ppm)	52.56 ab ± 3.19	48.39 bc ± 2.84	83.91 bc ± 5.56	74.47 b ± 5.56	24.40 b ± 2.3	24.79 b ± 2.0	1.39 ab ± 0.09	1.49 ab ± 0.09
	LSD	10.55	7.88	23.06	21.35	4.57	4.11	0.16	0.15

Mean of three replications (n = 3) ± SE and variants having same alphabets are not statistically significant at *P˂ 0.05*. Main factors and interaction are significant at *P˂ 0.01*. By using the LSD of the interaction between growth regulators and thermal regimes, lettering is done separately for each thermal regime.

Leaf chlorophyll were significantly reduced by high temperature regimes (April and May sown crops) during both years of study (thermal regimes × regulators *P* < 0.05) (Tables 3–4 and Fig. 3d–f). Further, the difference in leaf chlorophyll contents and net photosynthetic rate of the plant under supra and sub thermal regimes was also significant. All the growth substances improved leaf chlorophyll content than water treated plants. For example, 41% and 8% higher chlorophyll a content (averaged across of three reproductive/growth stages during two years of study) were observed in leaves treated with hydrogen peroxide under high-supra and medium-sub-optimal thermal regimes, respectively, than water-sprayed plants. Salicylic acid had no significant effect on chlorophyll content under all thermal regimes. Thermal regimes and growth regulators showed a significant two-way interaction (*P* < 0.05) for Pn, number of sympodial branches per plant and boll weight (g). Leaf Pn was equally affected by heat during all three developmental stages (squaring, flowering and boll formation). High temperature regimes (April and May sowing) caused 47% reduction in leaf Pn of water treated plants. Hydrogen peroxide increased leaf Pn by 29% under high temperature regimes (averaged across three growth stages and two years of study).

**Table 4:** Effect of growth regulators and thermal regimes on chlorophyll b contents and net photosynthetic rate-Pn (µ mol m^−2^ sec^−1^) in field conditions during 2012 and 2013.

Thermal regimes	Regulators	Chlorophyll b 2012	Chlorophyll b 2013	Pn 2012	Pn 2013		Fiber fineness (micronaire) 2012	Fiber fineness (micronaire) 2013	Fiber length (mm) 2012	Fiber length (mm) 2013
Optimal regime	Control	0.58 a ± 0.045	0.57 a ± 0.043	20.5 ± 1.37 c	19.2 ± 1.22 c	April	4.0 b ± 0.20	3.4 b ± 3.0	22.5 b ± 2.1	22.1 b ± 2.0
	H_2_O_2_ (30 ppm)	0.61 a ± 0.051	0.57 a ± 0.048	26.6 ± 1.63 a	24.6 ± 1.78 a		4.6 a ± 0.26	3.9 a ± 3.7	26.5 a ± 2.4	26.3 a ± 2.4
	SA (70 ppm)	0.55 a ± 0.040	0.53 a ± 0.039	20.3 ± 1.20 c	18.6 ± 1.15 c		3.8 b ± 0.23	3.3 b ± 3.1	22.7 b ± 2.0	22.3 b ± 2.1
	MLE (30 times diluted)	0.56 a ± 0.044	0.54 a ± 0.041	23.5 ± 1.35 b	21.5 ± 1.44 b		4.5 a ± 0.22	3.9 a ± 3.8	26.6 a ± 2.3	26.2 a ± 2.5
	ASA (70 ppm)	0.55 a ± 0.039	0.52 a ± 0.037	23.6 ± 1.50 b	21.4 ± 1.35 b		4.6 a ± 0.24	3.9 a ± 3.6	25.4 a ± 2.2	25.5 a ± 2.3
Supra-optimal	Control	0.40 b ± 0.032	0.41 b ± 0.022	17.7 ± 1.01 c	25.5 ± 1.50 b	May	3.3 b ± 0.17	4.1 a ± 4.0	19.1 b ± 1.7	27.0 b ± 2.6
	H_2_O_2_ (30 ppm)	0.52 a ± 0.038	0.53 a ± 0.033	24.1 ± 1.60 a	28.5 ± 1.85 a		3.8 a ± 0.26	4.2 a ± 4.1	23.6 a ± 2.3	28.1 a ± 2.7
	SA (70 ppm)	0.36 b ± 0.026	0.35 b ± 0.024	17.4 ± 0.98 c	25.1 ± 1.60 b		3.2 b ± 0.17	4.3 a ± 4.2	19.5 b ± 1.8	27.3 a ± 2.5
	MLE (30 times diluted)	0.48 a ± 0.035	0.50 a ± 0.048	20.8 ± 1.30 b	23.8 ± 1.45 b		3.7 a ± 0.23	4.1 a ± 3.9	24.5 a ± 2.2	27.2 a ± 2.4
	ASA (70 ppm)	0.48 a ± 0.037	0.49 a ± 0.039	20.6 ± 1.26 b	23.5 ± 1.35 b		3.8 a ± 0.17	4.0 a ± 3.8	23.4 a ± 2.1	26.8 a ± 2.3
Sub-optimal	Control	0.51 a ± 0.043	0.55 a ± 0.045	28.2 ± 1.71 b	28.7 ± 1.80 b	June	4.0 a ± 0.18	4.2 a ± 4.2	26.1 a ± 2.5	27.5 a ± 2.6
	H_2_O_2_ (30 ppm)	0.55 a ± 0.049	0.59 a ± 0.051	31.4 ± 2.01 a	32.0 ± 1.90 a		3.8 a ± 0.23	3.8 a ± 3.5	26.2 a ± 2.4	26.4 a ± 2.2
	SA (70 ppm)	0.49 a ± 0.040	0.54 a ± 0.037	28.0 ± 1.55 b	28.0 ± 1.60 b		3.8 a ± 0.21	4.1 a ± 3.7	26.4 a ± 2.6	25.6 a ± 2.2
	MLE (30 times diluted)	0.50 a ± 0.037	0.54 a ± 0.040	26.7 ± 1.42 b	26.9 ± 1.50 b		3.7 a ± 0.20	3.9 a ± 3.9	26.2 a ± 2.3	27.8 a ± 2.7
	ASA (70 ppm)	0.47 a ± 0.035	0.52 ab ± 0.041	26.4 ± 1.47 b	27.1 ± 1.65 b		3.7 a ± 0.22	3.8 a ± 3.6	25.9 a ± 2.1	26.6 a ± 2.4
	LSD	0.052	0.058	2.85	2.10		0.42	0.41	2.43	2.35

Mean of three replications (n = 3) ± SE and variants having same alphabets are not statistically significant at *P˂ 0.05*. Main factors and interaction are significant at *P˂ 0.01*. By using the LSD of the interaction between growth regulators and thermal regimes, lettering is done separately for each thermal regime.

**Figure 4:**
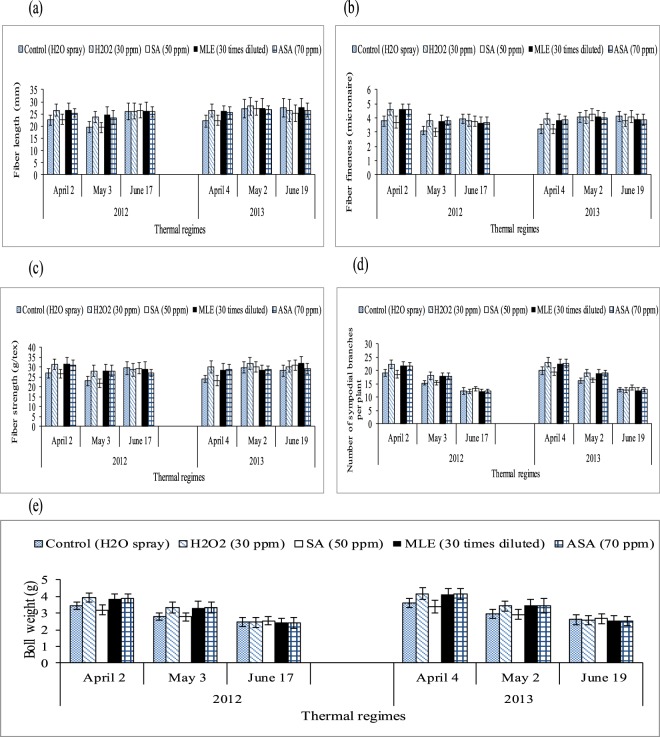
Effect of growth regulators and thermal regimes on (**a**–**c**) fiber quality components, (**d**) number of sympodial branches per plant and (**e**) boll weight under field conditions during both years of study. Values are the means ± SD (n = 3) and the bars indicate SD.

Number of sympodial branches per plant and averaged boll weight per plant (g) was reduced significantly under water treated plants of April and May sown crops than June sown crop. Among the growth regulators, H_2_O_2_, MLE and ASA increased number of sympodial branches per plant and averaged boll weight per plant (g) equally in April and May thermal regimes than salicylic acid and water treated plants (Table 5 and Fig. 4). On the other hand, hydrogen peroxide treated plants resulted 16.5% and 17.7% (averaged across two years of study) higher number sympodial branches under April and May thermal regimes respectively than water treated plants. Effect of different thermal regimes on fiber quality components are shown in Fig. 4a–c and Tables 4–5. June sown crop indicated more fiber length than May and April sown crop during the first year of study while in second year, both May and June sowing dates produced higher fiber length than the April sown crop. April and June sown crops produced higher fiber fineness and strength than May sown crop during 2012 while both May and June sowing dates represented more fiber fineness and strength during 2013 over the April sown crop

**Table 5:** Effect of growth regulators and thermal regimes on number of sympodial branches per plant and boll weight (g) in field conditions during 2012 and 2013.

Thermal regimes	Regulators	Fiber strength (g/tex) 2012	Fiber strength (g/tex) 2013	No. of sympodial branches (2012)	No. of sympodial branches (2013)	Boll weight (g) 2012	Boll weight (g) 2013
April	Control	27.5 b ± 2.6	25.2 b ± 2.4	19.1 b ± 1.23	20.0 b ± 142	3.4 b ± 0.30	3.6 b ± 3.4
	H_2_O_2_ (30 ppm)	31.5 a ± 3.0	30.1 a ± 2.9	22.4 a ± 1.64	23.1 a ± 1.81	3.9 a ± 0.36	4.2 a ± 4.0
	SA (70 ppm)	26.7 b ± 2.4	23.2 b ± 2.0	18.6 b ± 1.39	19.5 b ± 1.48	3.2 b ± 0.31	3.4 b ± 3.1
	MLE (30 times diluted)	31.6 a ± 2.9	28.8 a ± 2.6	21.8 a ± 1.51	22.7 a ± 1.70	3.8 a ± 0.37	4.1 a ± 3.9
	ASA (70 ppm)	31.2 a ± 2.8	28.8 a ± 2.5	21.7 a ± 1.43	22.6 a ± 1.59	3.9 a ± 0.38	4.2 a ± 4.2
May	Control	23.1 b ± 2.1	29.9 a ± 2.8	15.3 b ± 0.81	16.2 b ± 0.83	2.8 b ± 0.25	2.9 b ± 2.7
	H_2_O_2_ (30 ppm)	28.2 a ± 2.7	30.6 a ± 2.7	18.0 a ± 1.33	19.1 a ± 1.28	3.3 a ± 0.29	3.4 a ± 3.2
	SA (70 ppm)	22.1 b ± 2.0	30.1 a ± 2.6	15.4 b ± 0.82	16.5 b ± 0.84	2.9 b ± 0.26	2.9 b ± 2.6
	MLE (30 times diluted)	28.1 a ± 2.6	28.7 a ± 2.4	17.9 a ± 1.34	18.9 a ± 1.31	3.3 a ± 0.32	3.5 a ± 3.1
	ASA (70 ppm)	28.0 a ± 2.5	28.8 a ± 2.5	18.1 a ± 1.08	19.0 a ± 1.07	3.4 a ± 0.31	3.4 a ± 3.0
June	Control	29.6 a ± 2.9	29.3 a ± 2.8	12.4 a ± 1.38	12.8 a ± 0.74	2.5 a ± 0.22	2.6 a ± 2.5
	H_2_O_2_ (30 ppm)	28.7 a ± 2.7	30.4 a ± 2.9	12.2 a ± 1.70	12.7 a ± 0.87	2.4 a ± 0.20	2.6 a ± 2.4
	SA (70 ppm)	29.4 a ± 2.5	30.6 a ± 3.0	13.3 a ± 089	13.8 a ± 0.96	2.5 a ± 0.23	2.7 a ± 2.3
	MLE (30 times diluted)	29.3 a ± 2.8	30.0 a ± 2.7	12.1 a ± 0.83	12.6 a ± 1.01	2.4 a ± 0.22	2.5 a ± 2.2
	ASA (70 ppm)	27.2 a ± 2.4	29.2 a ± 2.5	12.3 a ± 0.70	12.8 a ± 0.87	2.4 a ± 0.21	2.5 a ± 2.4
	LSD	3.46	2.02	2.14	2.04	0.37	0.41

Mean of three replications (n = 3) ± SE and variants having same alphabets are not statistically significant at *P˂ 0.05*. Main factors and interaction are significant at *P˂ 0.01*. By using the LSD of the interaction between growth regulators and thermal regimes, lettering is done separately for each thermal regime.

For April-sown crop (2012), only H_2_O_2_significantly improved all fiber quality components of cotton but the effect of growth regulators e.g. H_2_O_2_, MLE and AsA was significant during 2013. For May-sown crop, H_2_O_2_, MLE and AsA showed significantly higher fiber quality parameters over water spray and salicylic acid during 2012, while during 2013 the effect of all regulators remained non-significant. All growth regulators showed non-significant results than control (water spray) in June sown crop during both study years under all quality components.

### Relationship between (MDA), fiber quality and boll weight under field conditions

The relationship between MDA, fiber quality parameters (Figs 7–11) and boll weight (Figs 10–11) was studied under regression analysis. The degree of association was found different for April, May and June sown crops. The relationship of MDA-fiber quality and MDA-boll weight was significant and negative under April and May thermal regimes during both years of study. The June thermal regime faced optimal temperature during both years of study demonstrating positive and non-significant association. May thermal regime during both years of study presented significant and negative association between MDA-quality parameters and boll weight. Under high temperature regimes (April and May), mean squares of regression were significant at *P* < 0.05 and were significant at 1% probability only at some places. The regression points were focused close to the regression line for negative association only in supra-optimal thermal regimes.

## Discussion

The first objective of these experiments was to understand how high temperature influences cotton growth and yield. Oxidative stress (ROS) due to abiotic stresses influences the integrity of chloroplast/photosynthetic machinery causing oxidation of cellular components, which are sensitive to heat ROS^23,24^. High temperature at three reproductive stages of cotton increased ROS e.g. lipid membrane peroxidation contents (MDA) which may affect the cell organelles as documented by^25^. A constricted balance between ROS and antioxidant enzymes is required^26,27^ but the stress conditions affected this balance between chloroplasts or mitochondria. The cotton plants were unable to protect cells from heat-induced damage of MDA under glass house (45/30 °C) and under high temperature sowing dates (April and May) of field study. The antioxidant enzymes i.e, SOD and CAT were up-regulated in cotton leaves but could not scavenge the ROS might be due to more stress at organelles. The present findings were supported by^4,28^. High temperature stress reduced chlorophyll contents and photosynthetic rate in tomato leaves^25^ which might be due to reduced CO_2_ fixation and photosynthesis process^29,30^. Relatively lower chlorophyll contents and net photosynthetic rate was observed in sub and supra-optimal thermal regimes of glass house and under high temperature regimes of field study at three reproductive stages of cotton crop might be due to higher oxidative stress. Medium and high temperature stress reduced number of sympodial branches and averaged boll weight (g) of cotton as documented by^31^ which might be due to hindrance in assimilates supply to developing bolls causing fruit abscission^32^. High temperature stress of glass house and field study decreased the fiber quality components (fiber fineness, length and strength) as reported by^33^ which might be due to reduction in photosynthesis as 90% of fiber is cellulose^34^.

To access the effect of various growth regulators such as H_2_O_2_, SA, MLE and ASA on plant defense, leaf physiology, yield and fiber quality components was the second objective of this study.

**Figure 5:**
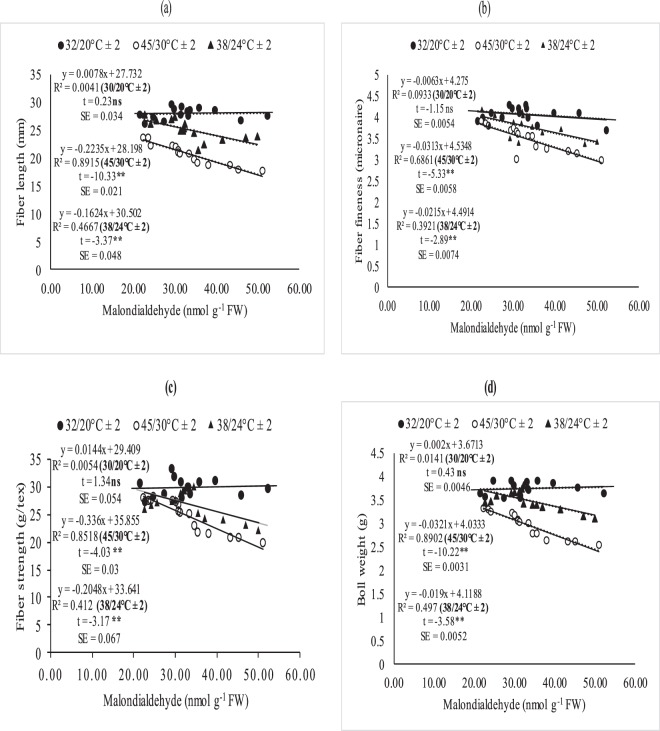
Association between malondialdehyde (nmol g^−1^ FW) and (**a**) fiber length, (**b**) fiber fineness, (**c**) fiber strength, (**d**) boll weight under glass house conditions. * and ** indicate significance at 5 and 1% levels, respectively.

Foliar spray of H_2_O_2_ under heat stress significantly enhanced SOD and CAT contents, reduced MDA contents and induced thermo-tolerance through capturing ROS^35,36^ found that H_2_O_2_ pre-treatment on cucumber leaves mitigated the heat stress effects, improved growth and reduced oxidative stress by protecting DNA structure. This might be due to strong signaling process of H_2_O_2_ which activates the plant defensive system against oxidative stress^37^. Similarly, foliar spray of *moringa* leaf extract and ascorbic acid increased antioxidants and decreased oxidative stress as documented by^38,39^. Hydrogen peroxide followed by *moringa* leaf extract and ascorbic acid improved chlorophyll contents and net photosynthetic rate under high temperature regimes of both field of studies. Hydrogen peroxide increased chlorophyll contents under high temperature regimes may be due to protecting the chloroplast from oxidative damage^40^. Similarly, ascorbic acid and *moringa* leaf extract improved chlorophyll contents under high temperature stress of mung bean and under salinity stress of wheat^39,41^ which might reduce senescence and increased photosynthetic pigments^42^. Hydrogen peroxide is a signaling molecule which might have activated the plant defensive system protecting the photosynthetic apparatus under heat stress conditions^43^. Similarly, *Moringa* leaf extract is rich with zeatine^44^ while ascorbic acid is most abundant antioxidant protecting the photosynthetic apparatus from oxidative stresses^45^ thus both may maintain the redox stat of photosynthetic apparatus. Apple yield and quality was improved when H_2_O_2_ was applied exogenously under normal conditions^43^ in contrary to high temperature regime of this study. Similarly, exogenous application of ASA and MLE increased yield and the related components under salinity and normal conditions of wheat^46,47^ as found in this study. The growth regulators (H_2_O_2_, MLE and ASA) increased fiber quality components under sub and supra-optimal thermal regimes of glass house and under high temperature sowing dates of field study (Figs 3a–c, 5a–c and Table 1). H_2_O_2_ has role in cell expansion, fiber development^48^ and is involved in secondary wall differentiation of cotton fibers^49–51^ as found in this study where H_2_O_2_ improved the fiber quality components under high temperature regimes. *Moringa* leaf extract is rich with cytokinins^17,52^ which may increase fiber quality components like IAA.

**Figure 6:**
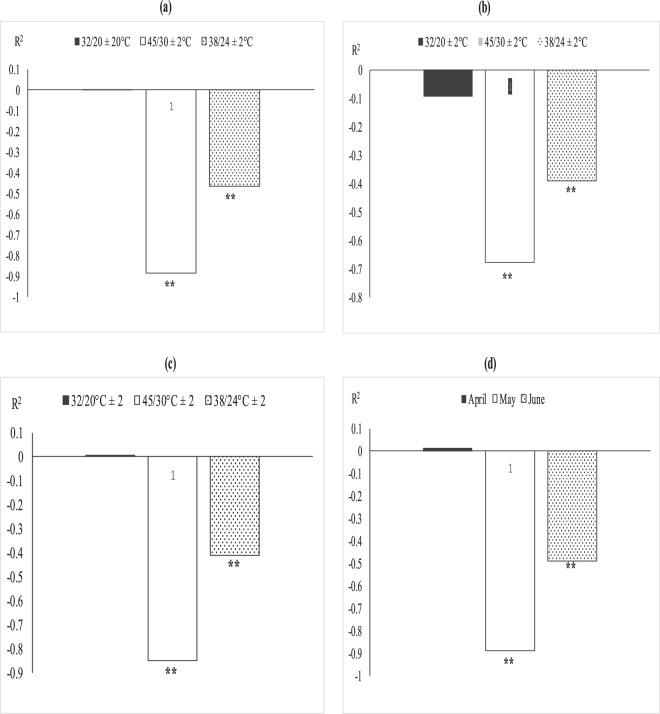
Difference of regression coefficient of (**a**) fiber length (**b**) fineness (**c**) fiber strength and (**d**) boll weight under glass house conditions. * and ** indicate significance at 5 and 1% levels, respectively.

**Figure 7:**
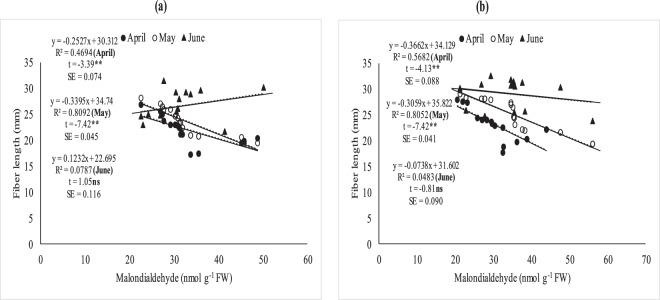
Association between malondialdehyde (nmolg-1FW) and fiber length under April, May and June sowing dates during 2012 (**a**) and 2013 (**b**) under field conditions. *and ** indicate significance at 5 and 1% levels, respectively.

**Figure 8:**
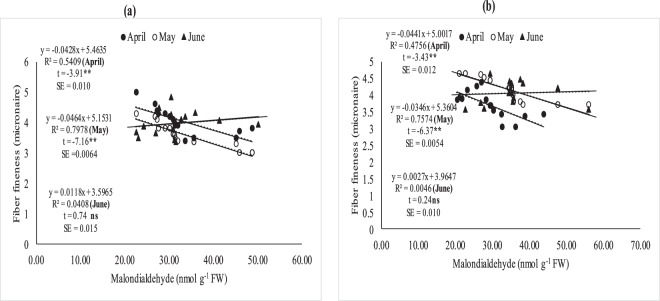
Association between malondialdehyde (nmolg-1FW) and fiber fineness in April, May and June sowing dates during 2012 (**a**) and 2013 (**b**) under field conditions. *and **indicate significance at 5 and 1% levels, respectively.

**Figure 9:**
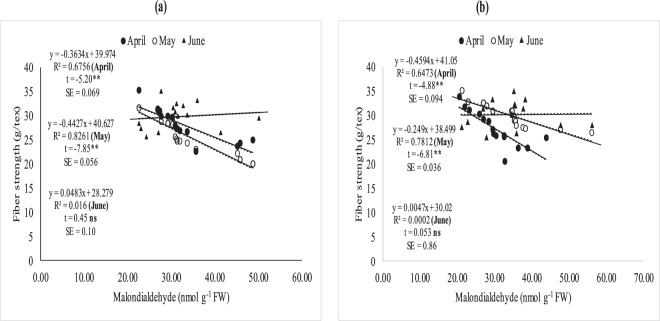
Association between malondialdehyde (nmolg-1FW and fiber strength under April, May and June sowing dates during 2012 (**a**) and 2013 (**b**) under field conditions. *and **indicate significance at 5 and 1% levels, respectively.

**Figure 10:**
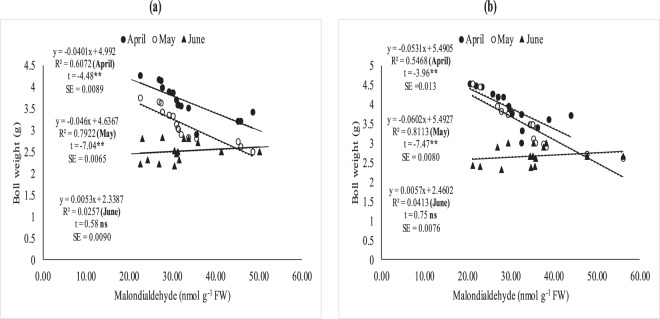
Association between malondialdehyde (nmolg-1FW) and boll weight under April, May and June sowing dates during 2012 (**a**) and 2013 (**b**) under field conditions. *and ** indicate significance at 5 and 1% levels, respectively.

**Figure 11:**
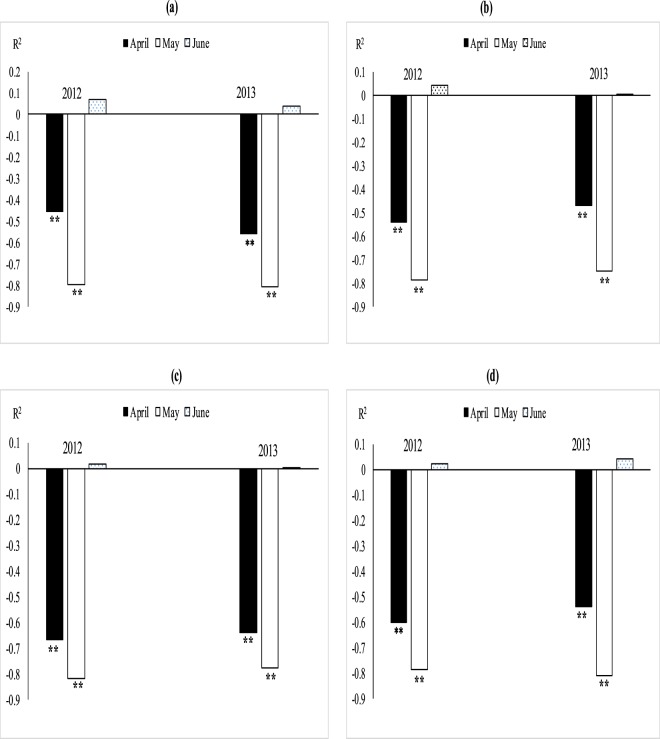
Difference of regression coefficient of (**a**) fiber length (**b**) fineness (**c**) fiber strength and (**d**) boll weight under field conditions. * and ** indicate significance at 5 and 1% levels, respectively.

## Conclusion

Cotton crop experienced significant reduction in yield and fiber quality when exposed to high temperature conditions during reproductive stages. This yield reduction was the result of lower number of sympodial branches and boll weight, which was associated with impaired leaf photosynthesis in heat stressed plants. Various growth regulators specifically H_2_O_2_ (followed by ASA and MLE) ameliorated heat-induced damage to cotton yield. These growth regulators also up-regulated antioxidant enzymes (SOD and CAT) activities, improved leaf chlorophyll and photosynthetic efficiency of cotton crops under medium and high temperature regimes.
