# Author Correction: Exogenously applied growth regulators protect the cotton crop from heat-induced injury by modulating plant defense mechanism

**DOI:** 10.1038/s41598-018-38076-3

**Published:** 2019-02-27

**Authors:** Muhammad Sarwar, Muhammad Farrukh Saleem, Najeeb Ullah, Muhammad Rizwan, Shafaqat Ali, Muhammad Rizwan Shahid, Saud A. Alamri, Mohammed Nasser Alyemeni, Parvaiz Ahmad

**Affiliations:** 1 Agronomic Research Institute, Ayub Agricultural Research Institute, Faisalabad, Pakistan; 2 Department of Agronomy, University of Agriculture Faisalabad, Faisalabad, Pakistan; 3 Queensland Alliance for Agriculture and Food Innovation, Centre for Plant Science, The University of Queensland Wilsonton Heights, Toowoomba, QLD 4350 Australia; 4 Department of Environmental Sciences and Engineering, Government College University Allama Iqbal Road, 38000 Faisalabad, Pakistan; 5 Institute of Soil and Environmental Sciences, University of Agriculture, Faisalabad, 38000 Pakistan; 6 Department of Botany and Microbiology, College of Science, King Saud University, Riyadh, Saudi Arabia; 7 Department of Botany, S.P. College, Maulana Azad Road, Srinagar, Jammu and Kashmir 190001 India

Correction to: *Scientific Reports* 10.1038/s41598-018-35420-5, published online 20 November 2018

This Article contains typographical errors in the Acknowledgements section.

“The authors would also like to extend their sincere appreciation to the Deanship of Scientific Research at King Saud University for its funding to the Research Group number (RG-1435–014).”

should read:

“The authors would also like to extend their sincere appreciation to the Deanship of Scientific Research at King Saud University for its funding to the Research Group number (RGP-199).”

